# The role of Mitofusin-1 and Mitofusin-2 in periodontal disease: a comprehensive review

**DOI:** 10.3389/froh.2025.1540178

**Published:** 2025-01-17

**Authors:** Sudhir R. Varma, Omar H. A. A. Ani, Jayaraj K. Narayanan, Asok Mathew

**Affiliations:** ^1^ Department of Clinical Sciences, Ajman University, Ajman, United Arab Emirates; ^2^ Center for Medical and bio-allied health Sciences Research, Ajman University, Ajman, United Arab Emirates; ^3^ Department of Basic Sciences, Ajman University, Ajman, United Arab Emirates

**Keywords:** Mitofusin-1, Mitofusin-2, periodontal disease, mitochondrial dynamics, oxidative stress

## Abstract

Periodontal disease is a widespread chronic inflammatory state influencing the supporting anatomy of the teeth, distinguished by oxidative stress, progressive bone loss, and tissue damage. Recent articles have highlighted the significance of mitochondrial dynamics, mainly Mitofusin-1 (MFN1) along with Mitofusin-2 (MFN2), inflammation regulation, tissue homeostasis, and in cellular function. The aim of the current study is to comprehensively review including evaluate the roles of MFN2 and MFN1 in the pathogenesis as well as the progression of periodontal disease, foregrounding their effect on mitochondrial integrity, inflammatory pathways, and oxidative stress. Studies were selected depending on inclusion criteria based on the roles of MFN2 and MFN1 in periodontal disease and health. Data from chosen *in vivo*, clinical studies, and *in vitro* were synthesized. Outcomes indicate that MFN2 and MFN1 are important for preserving cellular function, mitigating oxidative damage, and mitochondrial fusion. Decreased levels of these proteins were related to elevated oxidative stress, inflammation, and increased mitochondrial dysfunction in periodontal tissues. The current comprehensive review shows the important roles of MFN1 along with MFN2 in inflammation regulation, cell survival, and mitochondrial dynamics within periodontal disease. The prospective for targeting MFN1 along with MFN2 in therapeutic policy is promising, presenting avenues for upgraded periodontal management and regeneration.

## 1 Introduction

The phrase “periodontal disease” invokes a group of inflammatory diseases that encounter the gingiva, alveolar bone, and periodontal ligament, tissues that reinforce teeth. The process starts with the escalation of bacterial biofilms (a complex microbial community) on the surface of the tooth, initially leading to gingivitis (1). If gingivitis is ignored, it can turn into periodontitis and cause deeper pockets in the gum, resorption of alveolar bone, and degeneration of connective tissues, which can result in tooth loss. This progress from gingivitis to periodontitis is a backscattering of the damaging outcome of inflammation that is preserved inside periodontal tissue (2).

In older populations, periodontal disease is quite common. A global survey reported that acute periodontitis influences roughly 10%–15% of the adult population worldwide, which makes it one of the most widespread chronic diseases (3). Additionally, its effects on oral health and periodontal disease have been linked to several systemic conditions, along with respiratory disorders, cardiovascular disease, and diabetes mellitus. This systemic consortium among periodontal health including systemic inflammation, develops when inflammatory mediators including harmful bacteria go into the bloodstream (4).

Periodontal disease results from an inflammatory response not properly managed in response to the biofilm. The age of matrix-degrading enzymes (e.g., MMPs), and pro-inflammatory cytokines (e.g., TNF-α, IL-1β) is the outcome of chronic inflammation that is created by immune responses to specific periodontal pathogens like *Porphyromonas gingivalis*. This results in tissue degradation and bone loss (5). As a result of this microbial imbalance and inflammation, chronic oxidative stress occurs. This stress results in damage to the periodontal cells along with enhancing the inflammation, establishing a loop that accelerates the deterioration of tissue (6).

The mitochondria make ATP through the procedure of oxidative phosphorylation, which is crucial for the creation regulation of cellular redox states, reactive oxygen species (ROS), and apoptosis. Mitochondrial dysfunction along with oxidative stress, has been recognized as an important element in periodontal disease that contributes to persistent inflammation, and tissue loss (7). The fundamental procedure of mitochondrial fission and fusion, referred to as mitochondrial dynamics, is crucial for the preservation of mitochondrial function and quality. Defaced mitochondria can be restored by fusion, while defected mitochondria can be outlined and demeaned through fission. The inflammatory reactions along with periodontal tissue damage, can be escalated when these processes are dysregulated, leading to an elevated ROS generation (7, 8).

Additionally, immunological retaliation can be activated, and periodontal tissue inflammation can be exacerbated when mitochondrial dysfunction activates the release of damage-related molecular patterns (DAMPs) (9). The mitochondrial dysfunction has a systemic outcome in addition to exasperate local inflammation and may connect periodontal disease to conditions such as diabetes and cardiovascular disease, where oxidative inflammation and stress are the main factors (7).

**Figure 1:**
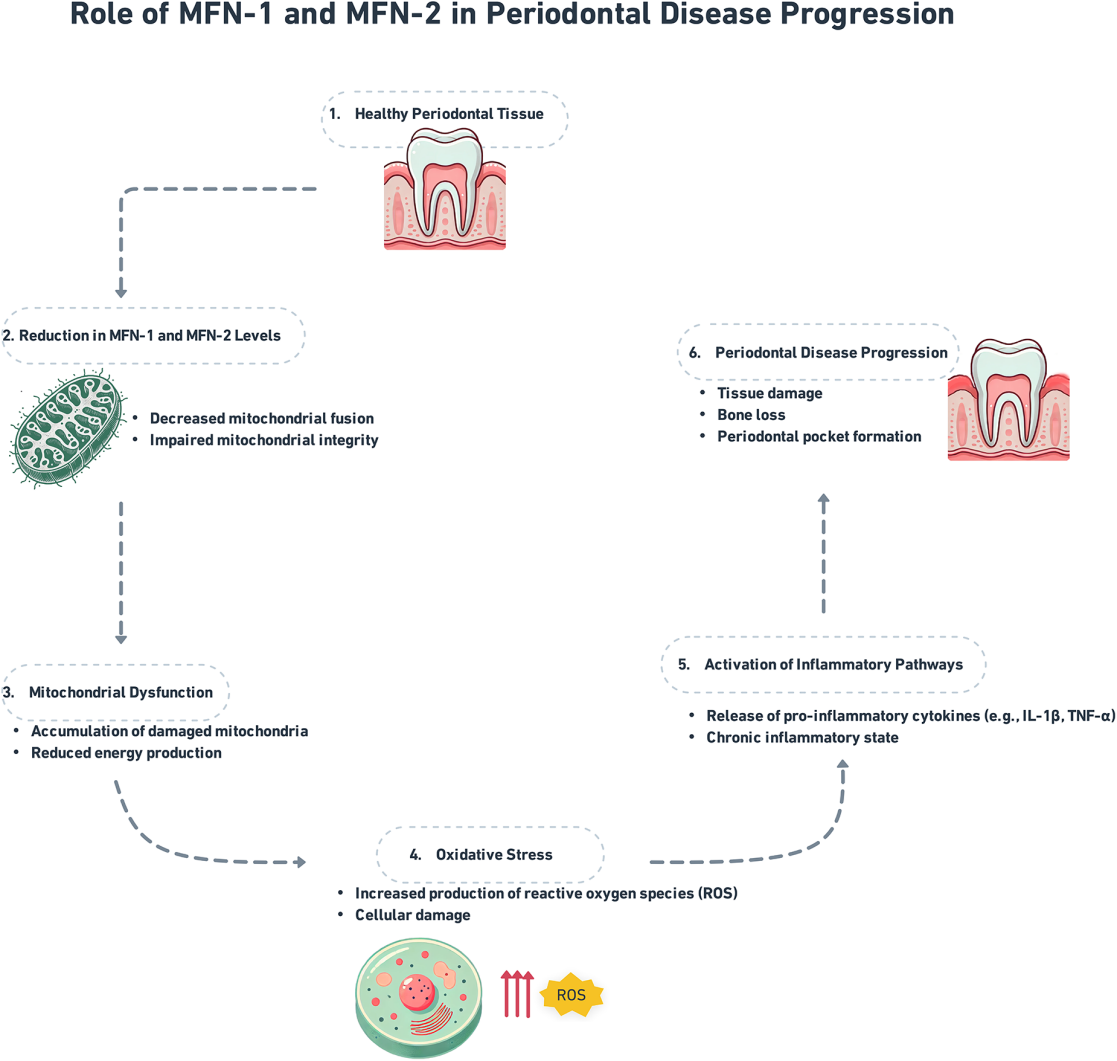
Role of MFN-1/2 in periodontal disease progression.

The GTPase proteins known as mitofusin-1 (MFN1) along with mitofusin-2 (MFN2) are reported on the exterior membrane of mitochondria as well as play an important role in managing mitochondrial fusion and are necessary to restore the functionality along with the integrity of the mitochondria (10). The mitochondrial fusion proteins MFN1 along MFN2 permit the contents of defected mitochondria to be repaired as well as healthy mitochondria to gather without the increase of dysfunctional mitochondria (11). Additionally, in its function in fusion, the MFN2 binds the mitochondria to the endoplasmic reticulum (ER), this is important for the regulation of lipid metabolism, calcium signaling, and apoptosis (12). The order of pro-inflammatory cytokine release is a single way, MFN2 modulates inflammation by this ER-mitochondrial interaction and also has ramifications for inflammatory signaling (13) (Figure 1).

MFN1 along MFN2 are both important for managing oxidative stress, this is a crucial grantor to the evolution of periodontal disease. Cellular resilience is promoted by Mitofusins, to stress-induced apoptosis as well as prevent immoderate ROS production through supporting mitochondrial health (14). These proteins if possible, are essential to the cellular procedure that underlies periodontal disease. Since oxidative stress is a vital reason for periodontal inflammation. Speaking about how MFN1 along MFN2 work in periodontal disease could give new intuition into pathogenesis as well as possible treatment approaches as they are important for preserving cellular stress, regulating inflammatory pathways, and mitochondrial integrity (15).

This comprehensive review aims to evaluate the part of MFN1 along MFN2 in the pathogenesis, including the progression of periodontal disease, focusing their effect on oxidative stress, inflammatory pathways, and mitochondrial integrity.

## 2 Structure and function of MFN-1 and MFN-2

The GTPase proteins MFN1 along MFN2, found on the external membrane of the mitochondria (Figure 1), are vital for mitochondrial fusion, preserving the integrity, including the functionality of the mitochondria (10). Fusion is important for facilitating the interchange of mitochondrial DNA, maintaining cellular energy production, and enhancing mitochondrial quality control by allowing damaged mitochondria to unite with healthier equivalents (11). In conditions such as periodontal disease, oxidative stress, and inflammation are frequent, MFN1 along MFN2 assist in preventing cell damage by managing mitochondrial integrity (16).

### 2.1 MFN1

The Mitofusin-1 (MFN1) gene is located on chromosome 3 in humans along with encoding the MFN1 protein. It is important for mitochondrial fusion, including dynamics (17, 18). MFN1 is a considerable GTPase within the dynamin-related protein family. It is distinguished by transmembrane domains that authorize its adjunct to the mitochondrial membrane along with GTPase domains that encourage energy-dependent conformational change. In terms of purpose, MFN1 along MFN2 work conjointly to mediate the external membrane fusion process, that maintains mitochondrial bioenergetics and shape (19). The procedure starts when MFN1 along MFN2 engage over neighboring mitochondria to generate a physical tether, draw 2 mitochondria closer together, and make a fusion previously. To help cells modify energy demands including stress, MFN1 encourages fusion and in turn, facilitates successful mitochondrial energy expenditure (11).

### 2.2 MFN2

The MFN2 protein is encoded by the MFN2 gene on chromosome 1 and shares structural attributes with MFN1, like transmembrane including GTPase domains (20). Moreover, MFN2 has additional activities past mitochondrial fusion. Apart from its function in mitochondrial dynamics, Mitofusin-2 is vital for the ER-mitochondria hitching (21). Lipid trade, cellular stress responses as well as calcium signaling all rely on this interplay between the mitochondria and ER. MFN2 manages inter-organelle transmission through this hitching, impacting cellular functions like cell cycle regulation along with apoptosis that goes beyond energy manufacture. According to studies, MFN2 can influence both pro- along anti-apoptotic pathways and help cells sustain under stressful situations. It is also involved in cellular apoptosis (22, 23). Periodontal disease is an inflammatory disease where a disparity of calcium, as well as apoptosis in cells, can exacerbate tissue damage, and MFN2's function in controlling ER-mitochondria transmission becomes more important (24, 25).

### 2.3 MFN1 and MFN2: similarities and differences

**Figure 2:**
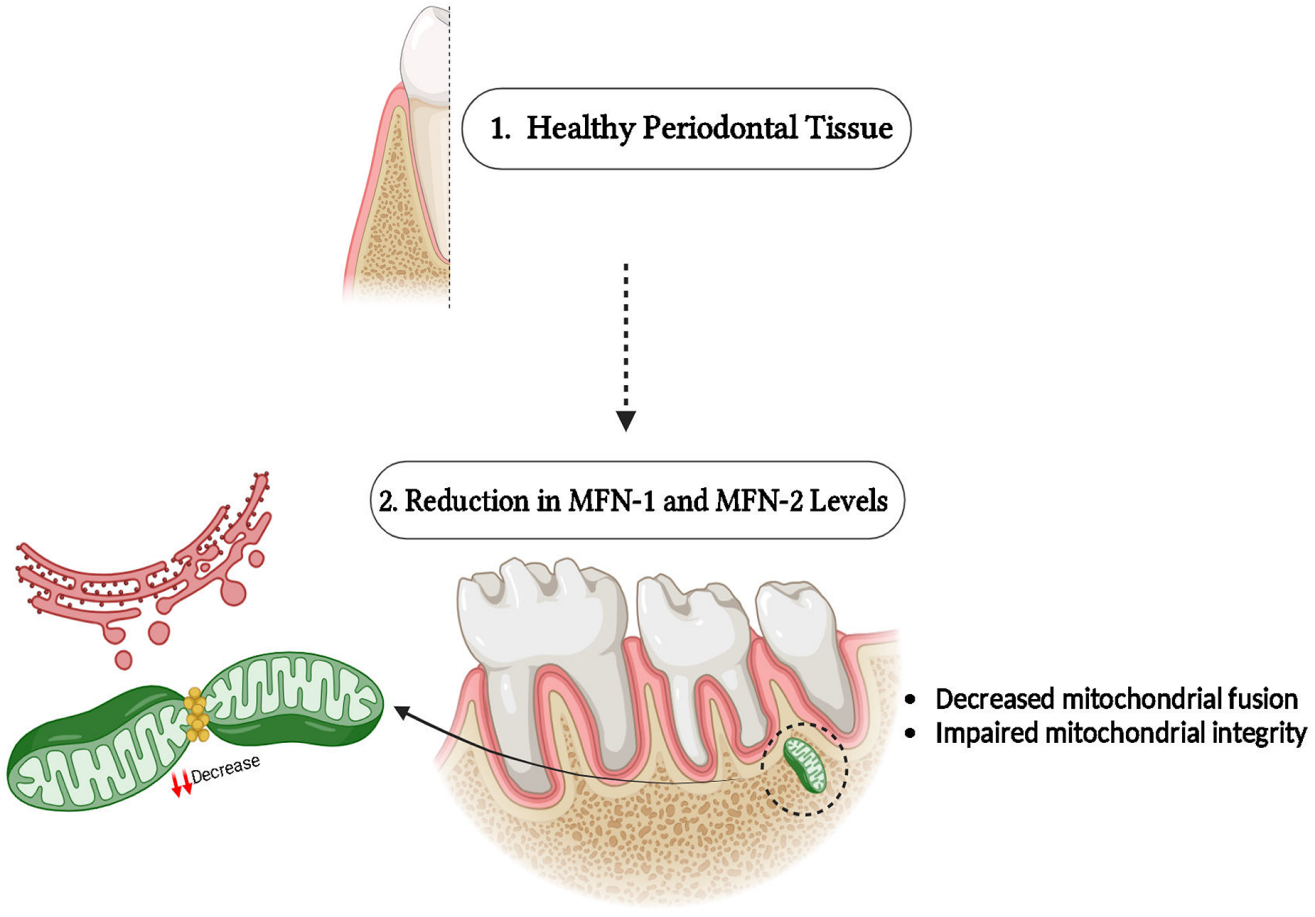
Role of MFN-1/2 in mitochondrial integrity.

MFN1 along MFN2 have different purposes throughout cellular procedures, even if they have a remarkable structural and functional overlap. The external mitochondrial membrane fusion pivots on both proteins and cooperates to preserve mitochondrial integrity and shape (26). Although Mitofusin-1 is mainly in charge of operating mitochondrial fusion, Mitofusin-2 has a wider influence on inter-organelle interplay via ER-mitochondria addition, which permits it to regulate calcium apoptosis and homeostasis. In contrast to MFN1, which primarily affects inflammation as well as cell survival, the MFN2 mediates ER-mitochondria connections, bridging the gap between the dynamics of mitochondria and responses to cellular stress (27, 28). Overall, the MFN1 and MFN2 are therefore absolutely essential for mitochondrial quality monitoring and function. Their cooperative participation in fusion, together with MFN2's extra roles in cellular signaling and apoptotic control, emphasizes their relevance in disease processes when oxidative stress, mitochondrial malfunction, and inflammation are clearly present, as in periodontal disease (7, 29). Therapeutic approaches targeting the health of mitochondria in persistent inflammatory diseases must be based on a thorough understanding of the complex roles played by MFN1 and MFN2 in these biological processes (Figure 2).

## 3 Mechanistic role of mitofusins in periodontal disease

### 3.1 Role in oxidative stress and mitochondrial dysfunction

**Figure 3:**
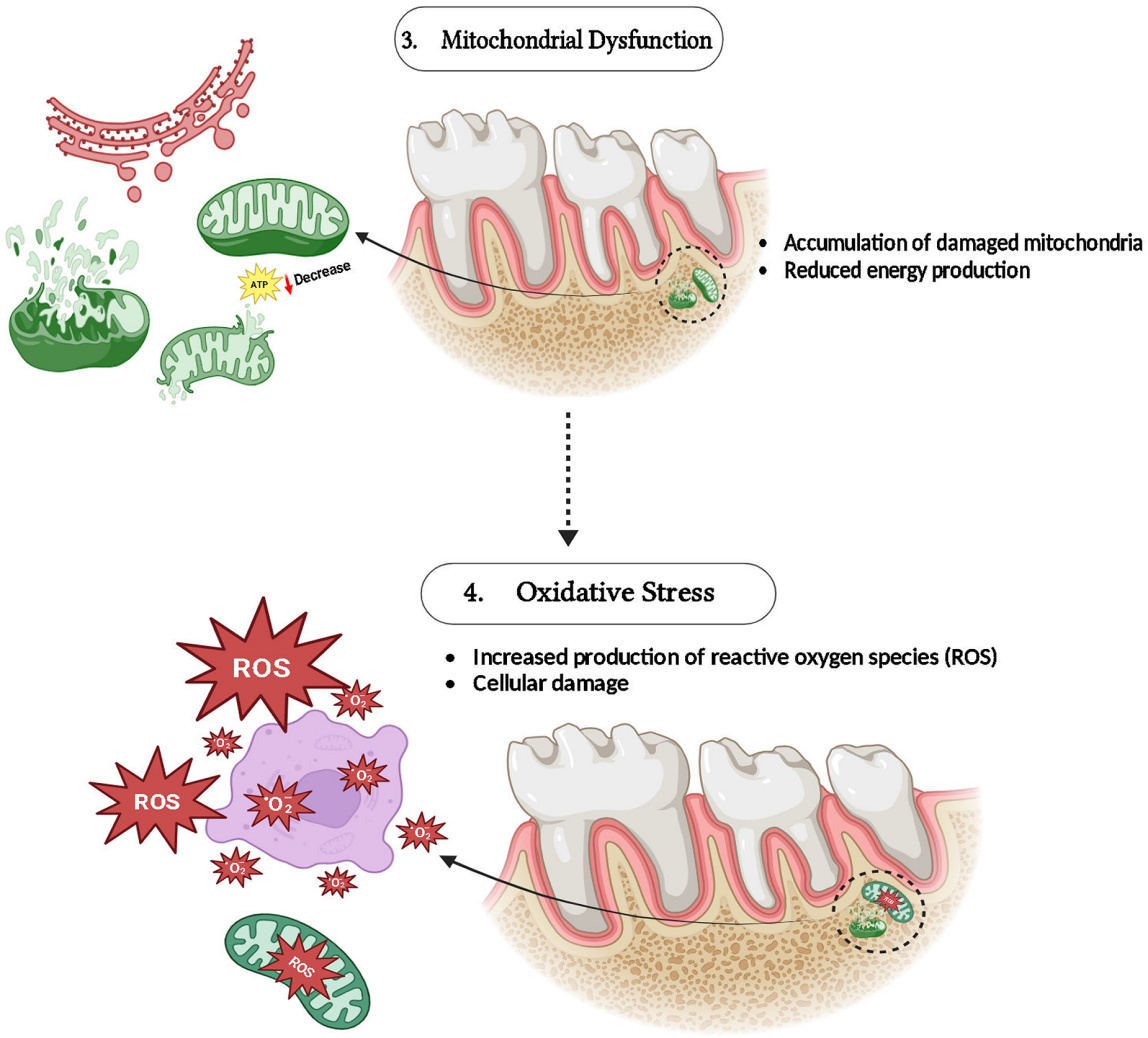
Role of MFN-1/2 in mitochondrial dysfunction and oxidative stress.

MFN1 along MFN2 are vital for preserving the probity of mitochondria as well as functionality by controlling mitochondrial fusion and a mechanism that reduces the outcomes of oxidative stress in the cells. Oxidative stress is a crucial factor in periodontal disease that leads to disease development, including tissue damage (30, 31). By promoting mitochondrial fusion, allowing damaged mitochondria to merge with ones that are healthy, MFN1 and MFN2 assist sustain mitochondrial function by dilution of defective components (32). ROS (Reactive oxygen species) generation is elevated whenever these mitofusins are reduced or malfunctioning, as is perceived in settings with important oxidative stress. By exasperating oxidative stress and destroying proteins, lipids, and mitochondrial DNA, this ROS excess speeds up the crash of periodontal tissue (33, 34) (Figure 3).

### 3.2 Influence on inflammatory pathways

The purpose of MFN1, along with MFN2 encompasses not just mitochondrial integrity and the regulation of inflammation, a crucial element in periodontal disease. Mitofusin-2 is important for attaching mitochondrial function besides inflammatory pathways by its association with ER-mitochondria hitching (7). The inter-organelle interactivity affects calcium signaling, and is important for activating inflammatory pathways. Studies indicate that MFN2 loss can diminish the connection between the mitochondria and ER, resulting in aberrant calcium signaling and elevated inflammatory responses (35). This disparity can exacerbate inflammation in periodontal disease and is a chronic inflammatory condition resulting by bacterial infections. It is considered that MFN2 can decrease tissue damage in periodontal disease, controlling the MAPK and NF-κB signaling pathways including regulating cytokine release, as a result dampening the inflammatory response (7).

### 3.3 Impact on cell death and apoptosis in periodontal cells

To preserve tissue homeostasis as well as facilitate cell turnover. The body employs apoptosis, which is called programmed cell death. Moreover, periodontal cells including gingival fibroblasts along with periodontal ligament cells, can speed up tissue loss including worsened periodontal disease, if they go through excessive apoptosis (36). Through their interconnection with apoptotic regulators including their preservation of mitochondrial health, MFN1 along MFN2 affects apoptosis. For instance, studies indicate that MFN2 may encourage cell death in cases of acute mitochondrial damage by pretending to be a pro-apoptotic agent (37). In comparison, periodontal cells are secured from stress-induced apoptosis by MFN1 along MFN2, which achieves this by encouraging mitochondrial fusion and reducing oxidative stress. In periodontal disease, inflammation and oxidative stress drive cells to apoptotic pathways, aggravating tissue destruction, their volume to control apoptosis is significant (38).

### 3.4 Interactions with the host immune response and pathogens

*Porphyromonas gingivalis* including other periodontal pathogens are capable of affecting the host immune system along with interfering with mitochondrial function. Evidence shows that MFN1 along MFN2 modulates the immune reciprocation to numerous pathogens. Immunological dysregulation and Mitochondrial fragmentation can result, as an example, from *P. gingivalis's* capability to hinder mitochondrial fusion and in turn, decrease the function of MFN1 along MFN2 (25). Mitofusins commit to mitochondrial function, thus modulating the immune system's feedback by comestible mitochondrial integrity, which is vital for ATP synthesis needed for immune cell activity. MFN1 along with MFN2 modulates inflammatory signaling and controls ROS generation, so influencing the host immunological reaction to pathogens and may influence the course of periodontal disease and severity (7).

## 4 Mitofusins in periodontal healing and regeneration

### 4.1 Role in cell survival and tissue regeneration

**Figure 4:**
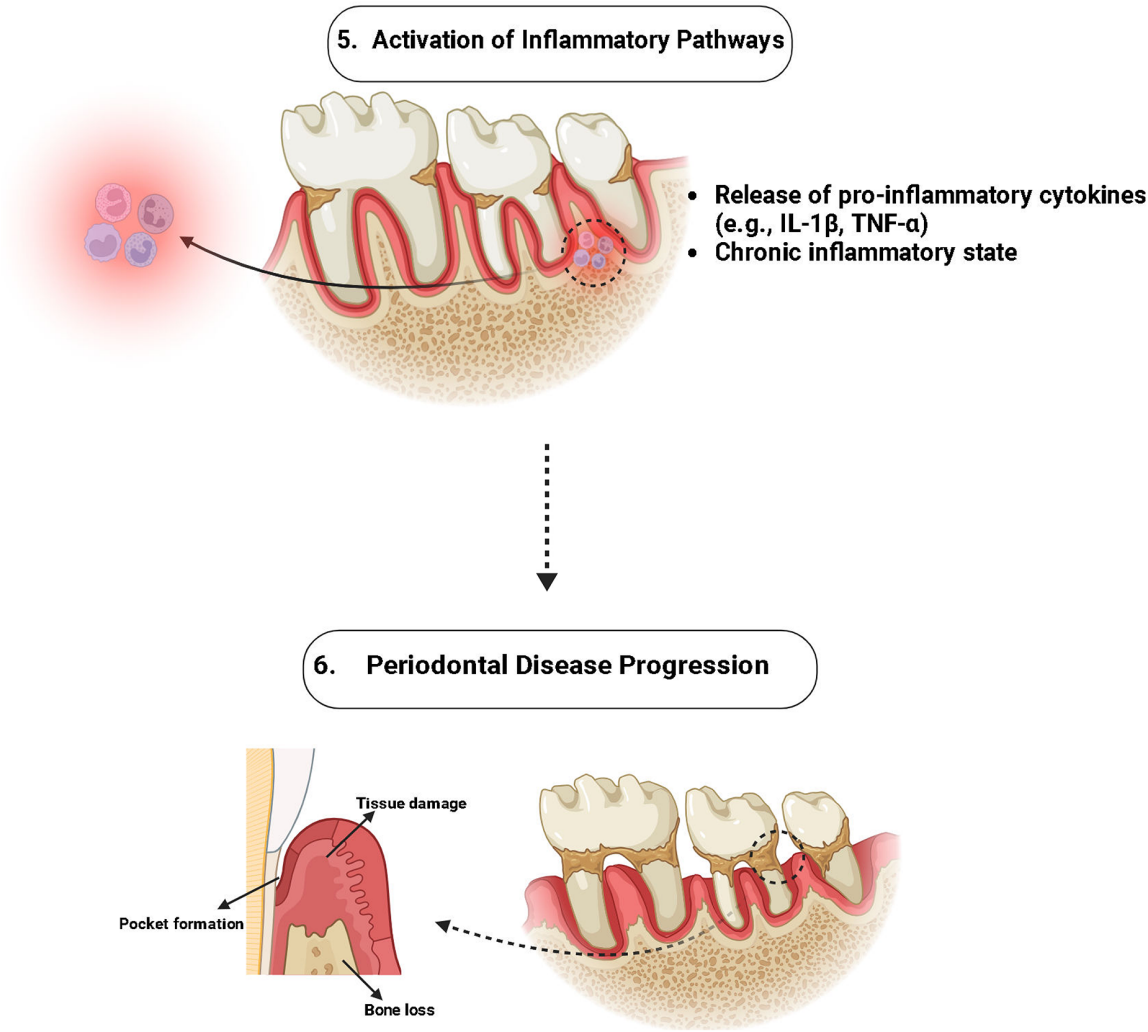
Role of MFN-1/2 in activation of pro-inflammatory cytokines.

The MFN1 along with MFN2 is vital for conserving mitochondrial integrity, and it is essential for tissue regeneration and cellular survival. These proteins ease mitochondrial fusion, allowing injured mitochondria to amalgamate with healthy equivalents, thus sustaining cellular function by continuing ATP synthesis and diluting defective elements (39). Inflammation and Oxidative stress are common in periodontal tissues, they frequently result in apoptosis and cell damage, which can hinder the healing procedure. Reducing mitochondrial fragmentation as well as lowering the creation of reactive MFN1, MFN2, and ROS, work to reduce these outcomes and elevate cell survival in inflammatory conditions (7) (Figures 3, 4).

In addition, research has exhibited that MFN2 mainly manages calcium homeostasis by hitching between the mitochondria and ER, a mechanism important for metabolism and cellular communication (40). Maintaining mitochondrial function is vital for the cellular repair procedure needed for periodontal healing. This is made possible using calcium management. Periodontal cells, like periodontal ligament cells and gingival fibroblasts, benefit from MFN1 along MFN2's protective results by controlling cellular stress and maintaining mitochondrial activity, increasing tissue resilience, and encouraging regeneration (15).

### 4.2 Influence on stem cell activity and differentiation

Both MFN1 along MFN2 can stimulate the growth of newly developed periodontal tissues by interrelating with the MSCs (mesenchymal stem cells) already present there. The mesenchymal stem cells play a key part in tissue regeneration because they can evolve into several cell varieties that are required for periodontal healing and fibroblasts along with osteoblasts. Mitochondrial dynamics, surrounding fission and fusion, are important for stem cell differentiation and functionality. MFN2 has been exhibited to facilitate osteogenic distinction by controlling mitochondrial bioenergetics, a vital element in periodontal repair and bone regeneration (38, 41).

Besides bioenergetic directive, MFN2 is associated with cellular signaling networks, which direct stem cell evolvement. Its part in ER-mitochondria communication is vital for maintaining intracellular calcium levels and is vital for starting differentiation signals in mesenchymal stem cells (42). The effectiveness of periodontal treatments focused on tissue regeneration by any means is diminished if dysregulation of Mitofusin-2 inhibits mesenchymal stem cell development (16). The regeneration possibility of MSCs is enhanced by Mitofusin-1 and Mitofusin-2, which regulates mitochondrial health and cellular stress responses. This helps with periodontal structure maintenance and tissue repair (7).

## 5 Clinical implications and therapeutic potential

### 5.1 Targeting Mitofusin-1 and Mitofusin-2 for therapy

The therapeutic possibility of targeting MFN1 along MFN2 for periodontal disease is ascribed to their purpose in mitochondrial fusion, control of inflammation, and cellular resilience. Gene therapy policy that enhances MFN1 or MFN2 utterance may ease the restoration of augmented energy generation, mitigate oxidative damage, and mitochondrial function in periodontal tissues (43). However, pharmacological activators of MFN1 along MFN2, and small compounds focused on encouraging mitochondrial fusion, have exhibited possible in preclinical investigations for safeguarding cells from inflammation and oxidative stress (44). Medications that balance mitochondrial dynamics along with decreased tissue degradation may be effective in the treatment of periodontal disease by activating mitochondrial fusion proteins (16).

### 5.2 Role of antioxidants and anti-inflammatory agents

Anti-inflammatory drugs and antioxidants may indirectly increase the action of MFN1 along MFN2 by alleviating oxidative stress in periodontal tissues. Increased oxidative stress disrupts mitochondrial dynamics. Hence, compounds like coenzyme Q10 (CoQ10) and N-acetylcysteine (NAC) may augment MFN1 along with MFN2 activity, therefore facilitating safeguarding periodontal cells and mitochondrial fusion from stress-related injury (38, 45). By reducing cytokine levels and maintaining mitochondrial integrity in periodontal tissue, anti-inflammatory drugs like curcumin may also assist modify MFN2's role in inflammation (46).

## 6 Materials and methods

### 6.1 Research questions

1. What is the current understanding of MFN1 and MFN2 roles in mitochondrial dynamics within periodontal disease?
2. How do MFN1 and MFN2 contribute to inflammation, oxidative stress, and tissue degradation in periodontal pathology?
3. What therapeutic potential exists for targeting MFN1 and MFN2 in the treatment and management of periodontal disease?

### 6.2 Search processing

A systematic search strategy was employed to identify relevant research articles across major scholarly databases, including PubMed, Scopus, Web of Science, and Google Scholar. Specific keywords and medical subject headings (MeSH) terms were used, such as “mitofusin-1,” “mitofusin-2,” “periodontal disease,” “mitochondrial dynamics,” “oxidative stress,” and “inflammation.” Boolean operators were applied to refine and expand the search; “AND” was used to combine terms, “OR” to broaden the search with synonyms, and “NOT” to exclude irrelevant studies (see Table 1 for detailed search strategies) (Table 1).

**Table 1:** Sources and searched strategies information.

Database	Search Strategies	Results
PubMed	(“Mitofusin-1” OR “MFN1”) AND (“Mitofusin-2” OR “MFN2”) AND (“Periodontal Disease” OR “Gingivitis”)	212
Scopus	TITLE-ABS (“Mitofusin-1” AND “Periodontal Disease” OR “Oxidative Stress”)	88
Web of Science	{[(AB = “Mitofusin-1”) OR (AB = “MFN2”)] AND AB = (“Periodontal Disease” OR “Inflammation”)}	134
Google Scholar	(“MFN1” OR “MFN2”) AND (“Periodontal Disease” OR “Mitochondrial Dysfunction”)	356
Total		790

### 6.3 Eligibility criteria and study selection

To ensure relevance and quality, specific inclusion and exclusion criteria were established.

#### 6.3.1 Inclusion criteria

• Studies focused on the role of MFN1 and MFN2 in mitochondrial dynamics and their implications in periodontal disease.
• Studies providing data on inflammatory processes, oxidative stress, or mitochondrial dysfunction related to periodontal health.
• Peer-reviewed articles, observational studies, clinical trials, and laboratory-based experimental studies.
• Articles in English to ensure consistent analysis and accessibility.

#### 6.3.2 Exclusion criteria

• Studies not directly addressing periodontal disease or irrelevant to MFN1 and MFN2.
• Publications focusing on non-periodontal diseases or non-mitochondrial aspects of cell biology.
• Studies in languages other than English or without full-text availability.

Titles and abstracts from the initial search were screened for relevance, followed by full-text retrieval for eligible articles. Two independent reviewers (authors A.B. and C.D.) conducted the screening and evaluated each study based on the defined inclusion and exclusion criteria.

### 6.4 Data extraction and quality assessment

The two reviewers independently extracted relevant data from the selected studies, which were then assessed for quality. The quality evaluation included assessing study design, sample size, and methodological rigor. The level of inter-reviewer agreement, measured by Cohen's Kappa coefficient (*K* = 0.80), indicated substantial agreement. Disagreements during study selection were resolved through consultation with a senior reviewer (author E.F.). Reference management software (EndNote version X9) was used to organize and manage citations.

### 6.5 PICOS requirements

**Population:** Studies focused on *in vitro* models, animal studies, and human populations involved in periodontal disease.

**Intervention:** Interventions or observations related to MFN1 and MFN2 influence on periodontal disease.

**Comparison:** Comparative studies examining different models or mechanisms of mitochondrial function within periodontal disease.

**Outcome:** Outcomes related to inflammatory markers, oxidative stress levels, and mitochondrial integrity in periodontal disease.

**Study Design:** No restrictions on publication year. Included peer-reviewed, full-text studies in English, such as observational, experimental, and review studies.

## 7 Results

### 7.1 Study selection

A total of 790 publications were identified from online databases, including PubMed (*n* = 212), Scopus (*n* = 88), Web of Science (*n* = 134), and Google Scholar (*n* = 356). No additional articles were found through manual search. After removing 478 duplicate records, 312 studies were retained for the title and abstract screening. Following the initial evaluation, 274 studies were excluded for not meeting the inclusion criteria, leaving 38 records for a detailed full-text review.

**Figure 5:**
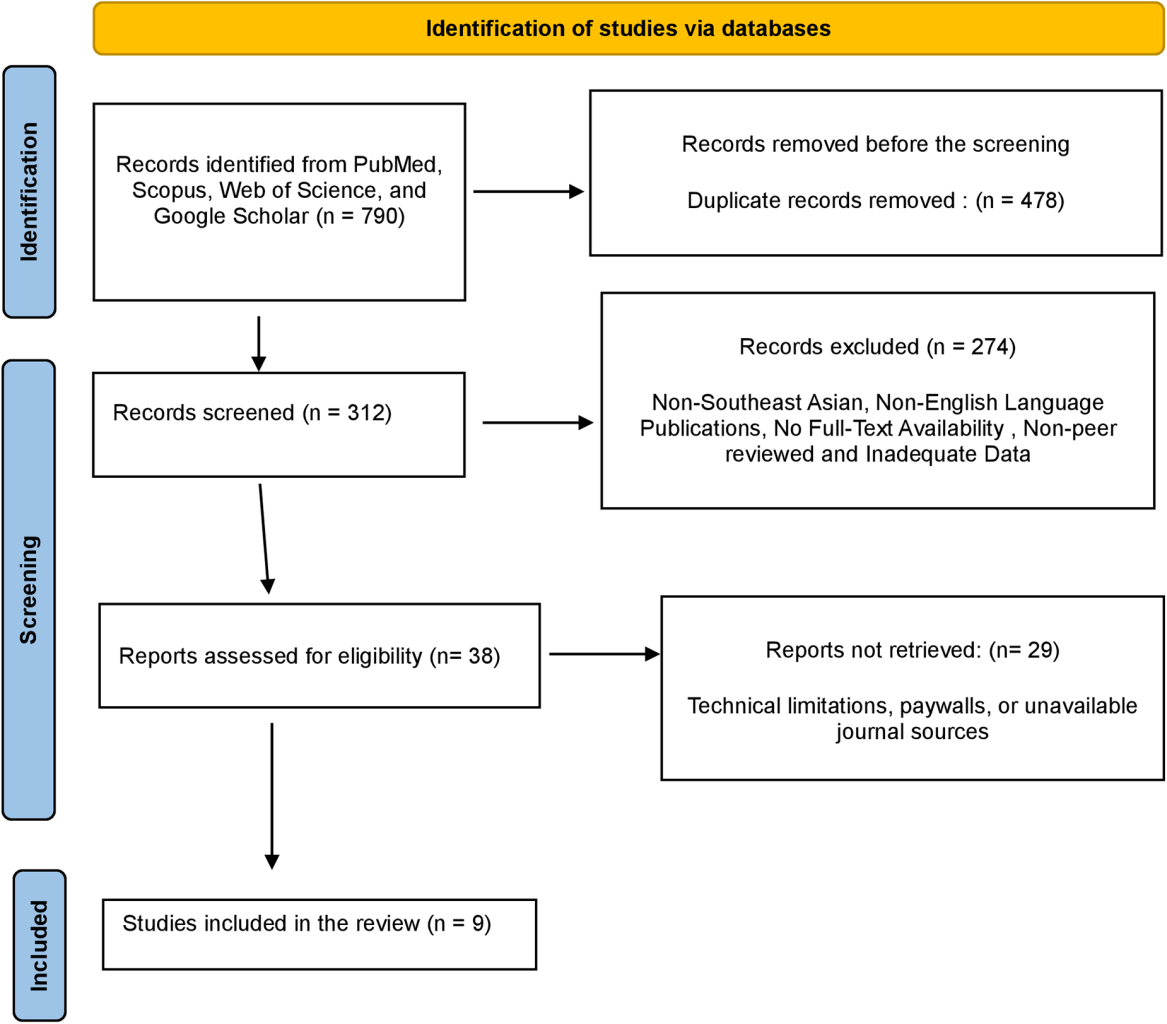
PRISMA flowchart demonstrating the selection process of articles retrieved from different web sources.

All 38 records were successfully retrieved for further assessment. However, 29 studies were excluded because their content did not align with the focus of the review, such as studies on non-periodontal diseases or research unrelated to mitochondrial dynamics. Ultimately, a total of 9 articles met the eligibility criteria and were included in the comprehensive review. The study selection process is depicted in the PRISMA flowchart (Figure 5).

### 7.2 Overview of included studies

The included studies in this review provide a comprehensive look at the roles of MFN1 and MFN2 in periodontal disease (Table 2), utilizing a variety of models, including *in vitro* and *in vivo* approaches. Key findings indicate that MFN1 and MFN2 levels are often reduced in periodontal disease conditions, correlating with increased oxidative stress, inflammation, and mitochondrial dysfunction. Several studies highlighted MFN2's unique role in ER-mitochondria tethering, influencing calcium signaling and inflammatory responses. Research also emphasized the impact of MFN1 and MFN2 expression on cellular survival and tissue regeneration, particularly in stem cell models. Overall, the evidence supports the hypothesis that modulating MFN1 and MFN2 activities could be beneficial in controlling oxidative stress and promoting periodontal tissue repair (Table 2).

**Table 2:** Summary of included studies on the role of Mitofusin-1 and Mitofusin-2 in periodontal disease.

Author and Year	Study Type	Objective	Sample/Model	Key Findings	Conclusion
Kırmızıgül Ö, A., et al., (15)	Invitro	To assess MFN1 and MFN2 levels in the gingival crevicular fluid (GCF) of patients with periodontal disease in comparison to healthy controls clinically.	GCF concentrations of MFN1, MFN2, tumor necrosis factor-alpha (TNF-α), and calcium (Ca), caspase-1.	The concentrations of MFN1, MFN2, Ca, caspase-1, and TNF-α were considerably lower in periodontal disease groups compared to healthy controls (*p* < 0.05). A positive correlation was seen among all assessed factors (*p* < 0.05).	The MERC protein MFN1 may contribute to the etiology of periodontal disease, as evidenced by its elevated levels in the gingival crevicular fluid of patients with periodontitis and gingivitis.
Vaseenon, S., et al., (47)	Invitro	This study compared inflammatory pulpal tissues to healthy pulp tissues in order to examine inflammation, oxidative stress, changes in mitochondrial dynamics, and cell death.	Human dental pulp	Inflamed pulp tissues had considerably reduced levels of MFN2.	Damage to the pulpal tissues, inflammation, oxidative stress, changes in mitochondrial dynamics, and cell death are all characteristics of irreversible pulpitis.
Aral, K., et al., (25)	Invitro	MFN1 and MFN2 levels were monitored in order to shed light on the possible role of mitochondria-endoplasmic reticulum interaction genes in the etiology of periodontal disease.	Primary human gingival fibroblasts	P. gingivalis, with or without F. nucleatum, shown a considerable upregulation of MFN1.	The expression levels of genes related to contact in human gingival fibroblasts may be variably dysregulated by F. nucleatum and P. gingivalis, either alone or in combination.
Deng, L., et al., (48)	Invitro	the novel function of MFN2 in myeloid-derived stem cell (MSC) energy consumption and osteogenic differentiation, which may lead to novel approaches to periodontal regeneration and repair of damaged alveolar bone.	Mesenchymal stem cells	The MFN2 deficiency facilitated the osteogenic differentiation of iPSC-MSCs and aerobic glycolysis with adequate oxygen, which raised the production of lactic acid and glucose consumption, as well as the activity of glycolytic enzymes and the expression of glycolytic genes.	The novel role of MFN2 in controlling MSC osteogenic differentiation and energy metabolism, which will offer a new therapeutic target and theoretical foundation for periodontal regeneration and alveolar bone repair.
Zhai, Q., et al., (49)	Invitro	To determine whether periodontal ligament stem cells (PDLSCs) express Mfn1.	Human periodontal ligament stem cells[	There was a significant difference in the expression levels of Mfn1 between H-PDLSCs and P-PDLSCs and H-PDLSCs + IL-1β (5 *μ*g/ml) groups (*P* < 0.05).	It is possible that inflammation enhances Mfn1 expression in PDLSCs while reducing the ability of P-PDLSCs to differentiate into osteogenic cells.
Zhai, Q., et al., (50)	Invitro	In investigate how periodontal ligament stem cells respond Mfn2 expression and endoplasmic reticulum (ER)-mitochondria coupling	Human periodontal ligament tissue	In comparison to H-PDLSC (0.99 ± 0.08), the expression level of Mfn2 was greater in P-PDLSC (1.46 ± 0.10).	Possible reduction in osteogenic differentiation of PDLSCs was accompanied by an increase in ER-mitochondrial coupling and Mfn2 expression in an inflammatory context.
Chen, Y., et al., (51)	Invitro	To investigate into whether mitochondrial malfunction contributes to the apoptosis of human PDLCs (hPDLCs) caused by OS and whether mitochondrial dynamics regulate the apoptosis of hPDLCs caused by OS.	Periodontal tissue	Hydrogen peroxide markedly enhanced mitochondrial fission by reducing the expression of Mfn1 and Mfn2.	The apoptosis of hPDLCs generated by oxidative stress may be mediated through a mitochondria-dependent mechanism.
Franco, A., et al., (52)	*in vivo*	To investigate the mechanisms of mutant MFN2 malfunction in CMT2A and to elucidate the links between mitofusin activator pharmacodynamics and disease response	Mice	This study examined the effects of MFN2 catalytic activity and allostery on defective mitochondrial fusion and neural transport in relation to Charcot-Marie-Tooth disease type 2A, an incurable peripheral neuropathy brought on by MFN2 mutations.	The findings mechanistically relate the repair of mitochondrial defects to the *in vivo* reversal of neurodegeneration in mouse CMT2A, and mitochondrial fusion and motility to the relaxed MFN2 protein shape.
Qin, S.-L., et al., (53)	*in vivo*	The purpose of the study was to describe alterations in mitofusin-1 (Mfn1) expression and determine whether any reported mitochondrial damage is linked to elevated oxidative stress.	Mice	Mfn1 protein and mRNA expression was evidently decreased in the renal tissues of rats with chronic fluorosis, but Fis1 expression was increased.	Chronic fluorosis can cause aberrant mitochondrial dynamics and altered kidney shape in rats. This condition may be caused by a high amount of oxidative stress.

## 8 Discussion

The included studies underscore the significant role that MFN1 and MFN2 play in mitochondrial dynamics and their implications in periodontal disease. Kırmızıgül et al. (2024) (15) demonstrated that the levels of MFN1 and MFN2 in GCF were notably lower in patients with periodontal disease compared to healthy controls, suggesting that reduced expression of these proteins correlates with impaired mitochondrial function and tissue health. This aligns with the concept that MFN1 and MFN2 are central to mitochondrial fusion, maintaining mitochondrial integrity and reducing oxidative stress, crucial for cellular survival (51).

Similarly, Aral et al. (2021) (25) found that exposure to Porphyromonas gingivalis led to significant upregulation of MFN1 in primary human gingival fibroblasts, suggesting that mitochondrial dynamics may be differentially regulated in response to bacterial infection. This distinctive regulation hinted that MFN1 along MFN2 contribute to compliant reciprocation within the inflamed periodontal environment. Moreover, Zhai et al. (2018) (49) transverse the expression of MFN1 along MFN2 in PDLSCs (periodontal ligament stem cells), disclosing that inflammation can increase MFN1 expression in periodontal ligament stem cells but decrease osteogenic potential. This advised that while mitofusins may counter inflammatory signals, their purpose could be circumstantial, influencing cellular procedure differently depending on the tissue type and inflammatory status. Deng et al. (2022) (48) supported these outcomes by reporting that MFN2 deficiency exhilarates the osteogenic differentiation of MSCs (mesenchymal stem cells) while elevating glycolytic activity, recommending that Mitofusin-2 plays a complex part in differentiation pathways and energy metabolism. Mitofusin-2 might balance mitochondrial dynamics and energy consumption, which is critical for cellular adaptation through periodontal tissue regeneration.

MFN1 among MFN2 significantly mitigates oxidative stress as well as inflammation and is censorious in periodontal disease. Chen et al. (2019) (51) reported that oxidative stress prompted by hydrogen peroxide induces reduced MFN1 along MFN2 expression in hPDLCs (human periodontal ligament cells), resulting in increased apoptosis and increased mitochondrial fission. This focused on how decreased mitofusin levels cause mitochondrial fragmentation, cellular damage processes, and increased ROS production that provide periodontal tissue degradation. Vaseenon et al. (2023) (47) reported their findings by disclosing that inflamed pulp tissues had remarkably lower MFN2 levels, showing that inflammation can damage mitochondrial fusion. This damage could amplify inflammation and oxidative stress, inducing tissue breakdown and cellular death. A study by Zhai et al. (2018) (49) on Mitofusin-2 in periodontal ligament cells showed that elevated Mitofusin-2 expression in inflammatory scenarios is associated with elevated ER-mitochondria coupling, and may regulate intracellular signaling and cytokine production. These findings recommend that while Mitofusin-2 can help arbitrate stress responses, its arbitrate may shift to promoting tissue damage and inflammation when dysregulated or overexpressed.

Kırmızıgül et al. (2024) (15) also reported positive correlations among MFN1, MFN2, along with inflammatory markers like caspase-1 and TNF-α in GCF. This shows that mitofusins may be incriminated in modulating inflammatory retaliation by mitochondrial pathways, striking complete periodontal tissue health. These findings imply that MFN1 along with MFN2 might enact regulators in the balance among contributing to inflammatory damage and maintaining cellular homeostasis when dysregulated.

The study of MFN1 along with MFN2 as a therapeutic quarry, offers a promising perception of periodontal disease care. Deng et al. (2022) (41) focused on the potential of regulating MFN2 to further optimize energy metabolism and osteogenic differentiation, which could promote bone repair and periodontal regeneration and periodontal regeneration. This proposes that therapies focused on enhancing Mitofusin-2 function could energize tissue regeneration including counteract the damaging outcomes of inflammation. The study of Chen et al. (2019) (51) designates that selected mitochondrial dynamics to preserve MFN1 along with MFN2 levels could prevent apoptosis and mitigate oxidative stress in periodontal cells. This shows the chance of utilizing pharmacological agents to encourage mitochondrial fusion as a therapeutic strategy. The research by Franco et al. (2022) (52) on deviant MFN2 in Charcot-Marie-Tooth disease exhibits that pharmacological activators of Mitofusin-2 can repair mitochondrial function including reverse neurodegenerative upshot *in vivo*. Extrapolating this proceed towards to periodontal disease, related pharmacological interventions could be investigated to increase mitofusin function, therefore reducing tissue degeneration as well as promoting healing.

In Addition, anti-inflammatory agents and antioxidants that help mitochondrial health could increse MFN1 along MFN2 activity, as proposed by Kumar, S., et al. (2024) (54). These agents could possibly synergize with selected therapies to reduce and maintain mitochondrial integrity and oxidative stress in periodontal tissues. The outcomes by Aral et al. (2021) (25), as well as Kırmızıgül et al. (2024) (15), also accentuate the prospective for gene therapy aimed at increasing mitofusin expression to prevent the mitochondrial dysfunction spot in periodontal disease.

Nevertheless, while the possibility of targeting MFN1 along with MFN2 is obvious, challenges endure. Qin et al. (2015) (53) exhibit that chronic conditions requiring oxidative stress, like fluorosis, reduces MFN1 expression and can disrupt mitochondrial dynamics. This hinted that the efficacy of selected therapies may differ based on independent patient conditions along with the expanse of mitochondrial damage. However, delivery mechanisms, long-term safety, and potential off-target effects of mitofusin targeting therapies should be exhaustively investigated prior to clinical application.

## 9 Limitations and future directions in research

This review has few limitations. It is constrained by the limited number of studies specifically focused on MFN1 and MFN2 in periodontal disease, with much existing research addressing broader mitochondrial dynamics or other conditions. The variability in study designs, such as *in vitro* vs. *in vivo* models, may also affect the generalizability and clinical applicability of the findings. Additionally, potential publication bias exists, as only peer-reviewed, English-language articles were included, potentially excluding relevant studies published in other languages either non-peer-reviewed sources. Labelling these limitations in further research could increase the understanding of MFN1 along with MFN2 roles in periodontal disease as well as support more strong therapeutic strategies.

Furthermore, targeting mitochondrial proteins such as MFN1 and MFN2 poses distinct problems. Gene therapy necessitates accurate delivery to periodontal tissues, and verifying the specificity of pharmacological agents to these proteins is intricate. Possible adverse effects, such as unexpected alteration of mitochondrial activity in many organs, continue to be a concern. Additional research is required to tackle these issues and formulate safe, tailored therapies for periodontal disease.

Future studies should concentrate on elucidating the precise mechanisms through which MFN1 along with MFN2 influence the progress of periodontal disease, particularly their functions in mitochondrial-endoplasmic reticulum interactions as well as the regulation of inflammation. *in vivo* investigations along with the creation of genetic models, such as MFN1 along with MFN2 knockout mice, are vital for elucidating their distinct parts in tissue regeneration and health. Clinical research examining the efficacy and safety of pharmacological compounds outline to enhance mitofusin activity may cause novel therapeutic alternatives. Furthermore, investigating the wider ramifications of mitofusins in many chronic inflammatory disorders could facilitate the advancement of holistic mitochondrial-targeted therapeutics. Confronting delivery obstacles and any adverse effects will be essential for the progression of these promising therapeutic approaches.

## 10 Conclusion

In conclusion, this comprehensive review highlights the critical roles of MFN1 and MFN2 in mitochondrial dynamics, inflammation regulation, and cell survival within periodontal disease. Reduced expression or dysfunction of these proteins contributes to oxidative stress, tissue damage, and impaired healing, underscoring their importance in periodontal pathology. The potential for targeting MFN1 and MFN2 in therapeutic strategies is promising, offering avenues for improved periodontal regeneration and management. However, further research is necessary to address knowledge gaps, refine therapeutic approaches, and ensure safe and effective clinical applications.
